# Older adults’ experiences of being screened for fall risk in a clinical setting: a focus group study

**DOI:** 10.1007/s41999-024-01056-0

**Published:** 2024-09-19

**Authors:** Nathalie Frisendahl, Patrik Karlsson, Stina Ek, Erika Franzén, Anne-Marie Boström, Anna-Karin Welmer

**Affiliations:** 1 Division of Physiotherapy, Department of Neurobiology, Care Sciences and Society, Karolinska Institutet, 141 83 Huddinge, 23100, Stockholm, Sweden; 2 Department of Neurobiology, Care Sciences and Society, Aging Research Center, Karolinska Institutet and Stockholm University, Stockholm, Sweden; 3 Unit of Epidemiology, Institute of Environmental Medicine, Karolinska Institutet, Stockholm, Sweden; 4 Women’s Health and Allied Health Professionals Theme, Medical Unit Occupational Therapy and Physiotherapy, Karolinska University Hospital, Stockholm, Sweden; 5 Research and Development Unit, Stockholms Sjukhem, Stockholm, Sweden; 6 Division of Nursing, Department of Neurobiology, Care Science and Society, Karolinska Institutet, Huddinge, Sweden; 7 Theme Inflammation and Aging, Medical Unit Aging, Karolinska University Hospital, Huddinge, Sweden; 8 Women’s Health and Allied Health Professionals Theme, Medical Unit Medical Psychology, Karolinska University Hospital, Stockholm, Sweden

**Keywords:** Community-living older adults, Feasibility study, Qualitative study, Primary healthcare, Prevention

## Abstract

**Purpose:**

There is a need for further research on older adults’ experiences of fall risk screening to improve health communication strategies. The purpose of this study was to describe and explore older adults’ experiences of being screened for risk of an injurious fall, using the first-time injurious falls (FIF) screening tool.

**Methods:**

A qualitative study with five focus group interviews was carried out including 17 older adults (11 women and six men, with a mean age of 77.4 years) who were recruited from two primary healthcare rehabilitation clinics in Sweden. Data were analyzed using reflexive thematic analysis.

**Results:**

The analysis generated one overarching theme, “Screening for fall risk promotes engagement by raising older adults’ awareness of their own abilities”, and four categories; “Screening may motivate to take action but can also create a false sense of security”, “Self-sufficiency is affected by the screening result and level of control over the environment”, “Easy-to-perform and helps to facilitate a discussion with the healthcare professional” and “Ideas of how FIF tool could be used in healthcare”.

**Conclusion:**

Older adults considered screening for fall risk to be meaningful insofar as it raises awareness of their own abilities and motivates them to prevent falls. On the other hand, a low fall risk could create a false sense of security, and lack of control over environmental factors related to fall risk could negatively impact their sense of self-sufficiency. They emphasized the need to receive support from healthcare providers and to be involved in care decisions if the screening indicates a high fall risk.

## Key summary points

**Aim:**

The aim of this study was to describe and explore older adults’ experiences of being screened for risk of an injurious fall, using the First-time Injurious Falls (FIF) screening tool.

**Findings:**

The participants considered screening for fall risk to be meaningful insofar as it raises awareness of their own abilities and motivates them to take action to prevent falls. On the other hand, a low fall risk could create a false sense of security, and lack of control over environmental factors related to fall risk could negatively impact older adults’ sense of self-sufficiency.

**Message:**

The results emphasize the need for continued support and assessment to older adults at high risk of injurious falls, and to actively involve patients in the planning of their care.

## Background

More than one-third of community-living older adults fall each year and about 10% of them require medical care as a result, making fall-related injuries in the older population a major public health problem [1]. Falls contribute to 5–10% of all emergency department visits among older adults, and contribute to serious consequences such as longstanding pain, disability, institutionalization, and increased mortality [1].

Having had a previous fall is shown to be the most prominent risk factor for falls [2]. Still, current screening methods tend to classify individuals without a history of falls as having a low risk of falling. However, it is noteworthy that a substantial number of falls may still occur among older adults without a previous history of falls [3]. Therefore, preventing or delaying that first fall through preventive efforts will have important effects on public health, as well as on the daily lives of older adults.

A new screening tool has been developed to predict the risk of first-time injurious falls in community-living older adults: the first-time injurious falls (FIF) screening tool [4]. The tool was based on previous research and constructed by combining previously identified risk factors from reviews and meta-analyses [5] and by weighting risk scores differently between men and women [6, 7]. The FIF tool, which is quick and easy to administer, consists of three self-assessment questions and a physical test (five-second standing balance on one leg with eyes open) (Appendix Table 3). The FIF tool has been validated in two cohorts in addition to the development cohort, and has shown high predictive values for first-time falls (Harrell’s C statistic ≥ 0.75 for both men and women in both the development and validation cohorts) over 5 years of follow-up [4, 8].

Understanding older adults’ thoughts about a screening tool is an important aspect of feasibility and implementation [9]. Patients’ experiences are becoming increasingly important when assessing the performance of healthcare systems worldwide [10]. Moreover, patients’ views on clinical quality and safety have become common practice when assessing the quality of care [11, 12]. Exploring patients’ needs and experiences further helps to give an understanding of how they perceive the care provided, as well as when and why it is experienced in a positive or a negative way. This may generate solutions to help solve challenges and further aid with the implementation of a patient-centered care pathway which, in turn, may result in reduced healthcare costs [13]. However, there is a need to further incorporate the patient and caregiver perspective into clinical practice guidelines and related health resources, since this, as of yet, has not been done on a consistent level, according to Montero-Odasso et al. [14].

A previous study demonstrated that older adults with high malnutrition risk commonly denied that they were at risk. The study states that this, in turn, may hinder a necessary behavioral change and the required uptake of recommended nutrition services [15]. This example illustrates the importance of understanding patients’ perspectives and experiences as part of improving health communication [15]. Another study described how older adults at a high risk of coronary heart disease postpone necessary changes until having a potential heart attack convinces them [16]. The case could be similar for fall risk, insofar as the signs of fall risk are not as tangible as the patients might need them to be. This indicates that there is a need for further research on the older population’s experiences of falls and screening for fall risk. We chose a qualitative approach with a manifest focus, as we were interested in getting a broader picture of the patients’ experiences that could not be obtained from a questionnaire. Therefore, the purpose of this study was to explore and describe older adults’ experiences of being screened for risk of an injurious fall using the FIF tool, thus aiding the FIF tool implementation process.

## Methods

### Design

Epistemologically, we assume that knowledge is subjective and constructed by social realities. Social reality is constructed through phenomena created by those living in a specific social context [17]. Based on the theoretical framework, a qualitative inductive approach was applied to this study [18, 19], using focus group methodology (i.e., interviewing groups of people collectively about a specific topic) [20, 21]. The focus group methodology was chosen because group interactions may empower and engage people, thus providing a rich understanding of the participants’ different perceptions of being screened with the FIF tool [20, 21]. Furthermore, the focus group methodology was considered a useful and suitable method since participants shared common experiences of some kind [20–22].

### Setting and participants

Participants were recruited from two primary healthcare rehabilitation clinics in two different locations in Sweden with different socioeconomic conditions. A purposive sampling was applied to this study [23], and all participants who had a booked appointment with their care provider and met the inclusion criteria (60 years of age or older, as well as speaking and understanding Swedish) received a letter from the care provider with information about the study. All participants who consented to be included in the study were screened with the FIF tool by their care provider at the primary healthcare rehabilitation clinic or at home between September 2021 and June 2022. The time between testing with FIF tool and the interview varied from 0 to 82 days, mean 30.3 [standard deviation (SD): 20.1 days]. A selection of those initially invited were contacted to ensure representation of a broad spectrum of participants from the target group (based on age and sex). They were then contacted by phone by the first author and asked if they wanted to participate in a focus group interview. Patients who agreed to participate were presented with different options regarding time of the interview and got to choose the proposed time best suited. Therefore, the constellations of the focus groups varied depending on patients’ attendance choice. Information on age, sex, reason for seeking primary healthcare, FIF tool score, and date of appointment was collected for all participants (Table 1). A total of 17 older adults (11 women and six men) consented to participate in five focus group interviews. The number of participants varied in each interview: four in the first one, two in the second, six in the third, three in the fourth and two in the fifth. Five of the participants had a prior fall for which they sought care. They were three participants that were included in an exercise class for balance training. Eleven participants declined to participate due to illness (*n* = 1), not being available (*n* = 7), or no longer wanting to participate in the study (*n* = 3). The mean age of the 17 participants was 77.4 years SD 10.6 years, with a range from 61 to 93.

**Table 1:** Descriptions of participants

Sex	Age	Cause of contact with primary care	FIF score 0-2p low risk 3 + p high risk	Time between tested with FIF tool and interview
Male	84	Rehab for quadriceps rupture	3 points	29 days
Female	64	Rehab for lower fracture	3 points	34 days
Female	69	Rehab for ankle fracture	1 point	50 days
Male	90	Back pain	5 points	62 days
Female	61	Assessment of physical aid needs	2 points	63 days
Female	64	Shoulder pain	1 points	81 days
Male	75	Exercise group	5 points	26 days
Male	83	Exercise group	3 points	26 days
Male	91	Exercise group	7 points	0 days
Female	72	Arthrosis	4 points	7 days
Female	83	Arthrosis	4 points	21 days
Female	93	Arthrosis	7 points	21 days
Female	73	Neck pain	5 points	14 days
Female	88	Vertebral compression	6 points	20 days
Female	78	Impaired balance	5 points	20 days
Female	84	Heart failure	2 points	28 days
Male	63	Cardiac rehabilitation	0 point	21 days

### Data collection

The data were collected through five focus group interviews conducted between November 2021 and June 2022 at the two primary healthcare clinics. All participants were community-living older adults who previously visited the outpatient rehabilitation clinic as patients, hence, the surroundings were familiar. Three interviews were conducted by the first and the second author, with one serving as moderator and the other as an observer. The observer took notes about aspects which could not be captured on the recordings, such as the interaction between the respondents. Two additional focus group interviews had only the first author as a moderator, without any observer. The interviews were conducted based on a semi-structured interview guide (Appendix Table 4) [22]. The interview guide contained several questions regarding thoughts about their experiences of being screened with the FIF tool and what the participants thought about screening for risk of an injurious fall. To create an interview atmosphere that fostered a sense of trust, the moderator and observer began each session by introducing themselves and describing their professional background and their role in the study. The opening question was “Can you describe what you associate with screening?”. Follow-up questions were then asked to delve deeper into the answers until the questions were fully explored. The interviews varied in duration between 18 and 42 min, depending on the number of participants and how in-depth the interviews turned out.

### Data analysis

A discussion was held between the moderator and the observer about their first impressions of what was perceived during the interviews, before continuing with the transcription as a first step in the analysis process. Recordings of the five focus group interviews were transcribed verbatim by the first author. Inductive qualitative content analysis was used as the method of text analysis because it provides a broad picture of the participants’ experiences [24, 25]. Initially, all the transcribed texts were read by the first and second author to enable them to familiarize themselves with the material. The analysis of the text then followed an inductive qualitative content analysis process [24, 25]. To ensure trustworthiness, more than one member of the research team took part in the analysis. Thus, the first author performed the first analytical steps, in which meaning-bearing units with similar content were extracted from the text and classified, condensed, coded, and categorized. The second author independently read and familiarized themself with two randomly selected interviews to validate the preliminary analysis performed by the first author. Both authors refined and relabeled the categories by discussing and comparing the emerging results and interpretations with the content of the original interviews until consensus was reached. The last author acted as a peer reviewer in the latter part of the analytical process and assessed whether the findings reflected the content of the interviews. Finally, an overall theme covering all five categories was developed. An example of the analysis process can be found in Table 2.

**Table 2:** Data analysis process

Meaning unit	Condensed meaning unit	Code	Category	Theme
Well, we’re mature women who go to balance and strength training. We’re all aware that we have an increased risk of falls? Varies, yes. So I don’t know if it’s good… Does it help to find out if you have a low risk of falls or a high risk of falls? Yes, perhaps a high risk of falls then, maybe I can prevent. And so if I have a low risk of falls, then I’ll stop and fall all over the place	We’re well aware that we have an increased risk of falls? Does it help to have a low or high risk of falls? Yes, perhaps a high risk of falls then, maybe I can prevent	A high risk of falls can be prevented	Screening may motivate to act but can also create a false sense of security	Screening for fall risk promotes engagement by raising awareness of older adults’ own abilities
Yes, that’s always good. It’s certainly a good thing to exercise and to stand on one leg every day. And everything, so that you notice that if you can’t, then there’s a greater risk of falling naturally. So that’s how it is	That’s always good. Good to stand on one leg every day. Notice that if you can’t, then there’s a greater risk of falling	Relevant to be alert	Screening may motivate to act but can also create a false sense of security	
I have a walker at home but I can’t use it much. There are small spaces, and there are thresholds and so on. It’s hard to get in, and it’s difficult. Going into the toilet. It doesn’t work	I have a walker at home but I can’t use it much. There are small spaces, there are thresholds. Going into the toilet, it doesn’t work	Despite aids, the home environment is difficult	Self-sufficiency is affected by the screening result and lack of control of the environment	
And what causes the fall. There’s a lot of ice on the streets, there’s like a paving stone that stands up so you trip on it. So it’s not just the fact that you’re prone to falling easily, it’s your surroundings that also cause you to fall Snow that hasn’t been swept away properly	There’s a lot of ice on the streets. There’s like a paving stone that stands up so you trip. It’s not just the fact that you’re prone to falling easily, it’s your surroundings that are also the cause. Snow that hasn’t been swept away properly	The affect of surroundings	Self-sufficiency is affected by the screening result and lack of control of the environment	
Yes, that was absolutely fine. Not hard at all. I got praise for having quite good balance. So that was absolutely good	OK, not hard at all. I got praise for having good balance. That was good	The balance test went well	Easy to perform and helps to facilitate a discussion with the healthcare professional	
Not unusual, no. There were questions, so it was just a matter of answering all the questions, so there was nothing strange. No	Not unusual. It was just a matter of answering. Nothing strange	No strange questions	Easy to perform and helps to facilitate a discussion with the healthcare professional	
I thought exactly about that, what the situation with your knees is. I thought about this test that you do. I thought it was simple. And I’m thinking spontaneously that if you were to do screening, I can think of at least two or three different things, including this matter of getting up, for example	It was simple. If you were to do screening, I can think of two or three different things, including this matter of getting up, for example	Several physical tests	Ideas of how FIF tool could be used in healthcare	
Yes, at a doctor’s appointment, for example. When you go to the doctor for an annual check-up, then they do that, a quick little test to start prevention	When you go to a doctor for an annual check-up, that they do a quick little test to prevent	Screening at doctor’s appointment	Ideas of how FIF tool could be used in healthcare	

## Results

### Overarching theme

#### Screening for fall risk promotes engagement by raising older adults’ awareness of their own abilities

The informants described screening for fall risk as meaningful and important since it may enable prevention of falls in the future by raising awareness of their own abilities. They described that they perceived falls as highly complex and that the screening promoted engagement and motivated them to act to further prevent falls in the future. However, the informants also described a feeling of lack of control even if they were to become more aware of the environmental factors and situations that could further increase the risk for injurious falls. They further described that receiving a low fall risk score may create a sense of false security. Lastly, they did not experience any difficulties performing the FIF tool and even provided suggestions about future uses of the tool in the primary healthcare.

The overarching theme was based on four different categories:

- Screening may motivate older adults to act, but can also create a false sense of security
- Self-sufficiency is affected by the screening result and level of control over the environment
- The FIF tool is easy to perform and helps to facilitate a discussion with the healthcare professional
- Ideas for how FIF tool could be used in healthcare

#### Screening may motivate older adults to act, but can also create a false sense of security

Informants described that they believed screening for fall risk could prevent falls and that preventive measures were important to avoid injurious falls. The informants described that screening for fall risk could give them an idea of their own abilities as well as an assessment of what is needed to prevent falls. Some informants said that the results were aligned with their own perceptions of their own abilities and that the results were almost perceived as being expected.

It is important to detect fear of falling early as the informants expressed how a fear of falling could increase their fall risk. If the informants had experienced previous falls and had a fear of falling, they expressed how they applied fall avoidance strategies, such as increased alertness and avoiding hasty movements or activities that could increase the risk of falls. Some informants expressed that they needed to learn how to fall safely by taking part in a course on fall techniques. They also described that walking aids reduced their concerns about falling. Additionally, they perceived it as being important to receive support from their healthcare professional regarding how to avoid falls if the FIF tool screening indicated a high fall risk.

“…you’re doing this [FIF tool screening], and then you get a high fall risk score. Subsequently, if you then find that out, there’s a high risk that you will fall. Then you have to get some tips or tools on how to avoid it.” (Interview 4).

Screening was generally perceived to be important, but some informants felt that they were too young to be screened since they did not feel that they were at risk of falling nor had experienced any previous falls. A screening result could also create a false sense of security if the test indicated a low fall risk. The informants thought that even a low risk of falling should be considered as a risk of falling, as to not instill a false sense of security.

“I agree with you that you shouldn’t, you should always assume that there is always a fixed risk of falls, whether it’s high or low. So, I can fall anyway.” (Interview 4).

#### Self-sufficiency is affected by the screening result and level of control of the environment

The informants argued that falls are a complex issue. They were aware of risk factors for falls and described how they often thought about these risks and their possible consequences. They reflected on how these thoughts about fall risks influenced their everyday life and daily behaviors. The informants described how the individual must be allowed to create changes to prevent falls without someone else deciding and/or giving advice (i.e. coming up with pointers), and a screening result could therefore help by providing the right conditions for this:

“Coming up with pointers, and doing this and that at home and removing carpets isn’t going to do anything. People my age, and all of us in general, we have our set ideas and thoughts and habits. That’s kind of how it is.” (Interview 5).

The informants described that when you receive the FIF tool results, or when a healthcare professional makes an assessment, it was easier to change your behavior to avoid falls. The result helps to show the informants where the problem could be in a more concrete way.

The informants described how falls were not only due to their own abilities, but were also caused by the environment. For example, difficulties outdoors and at curbs or on uneven surfaces, especially in winter. They described how the home environment was adapted according to their needs, but that it was difficult to avoid falls despite using aids. This was due to tight spaces and difficulties getting around indoors using aids. When talking about the probability that they would fall, the informants thought that a fall would be more likely to occur because of a lack of control over environmental factors rather than a problem with their balance.

“I have a walker at home but I can’t use it much. There are small spaces, and there are thresholds and so on. It’s hard to get in, and it’s difficult going into the toilet. It doesn’t work.” (Interview 3).

#### The FIF tool is easy to perform and helps to facilitate a discussion with the healthcare professional

When using the FIF tool, the informants generally felt that testing the new screening instrument went well. They described the FIF tool as a routine test composed of general questions and felt that the test was easy to carry out. The questions were also perceived as being relevant for a fall risk screening tool and not too sensitive to answer. Instead, the informants described that the questions had a neutral tone. However, the questions in the FIF tool and the one-leg balance test were sometimes difficult to remember afterwards. The informants also described how it could be hard to remember the results, and that it could be unclear whether they had received a result or it had gone unnoticed.

“There were no weird questions. It felt completely normal.” (Interview 1).

The informants described how they perceived the balance test to be the most important aspect of the screening instrument, as it gave a quick result about their own abilities. Further, they described how measuring the seconds standing on one leg, felt important and relevant. They felt confident performing the balance test together with their care provider as it was done in a safe environment.

“It was easy for me. Yes, I didn’t think standing on one leg was difficult at all.” (Interview 5).

The results that the informants obtained from the screening instrument were perceived as being meaningful. It was perceived as being important and also joyful to find out their results, whether or not the results indicated an increased risk of falling. Informants described that the FIF tool could facilitate a meaningful conversation and discussion with the care provider. It allowed the informants to discuss viable interventions regarding the results and their risk of falling.

#### Ideas for how the FIF tool could be used in healthcare

The informants perceived that only one round of screening was far too little to be able to make a fair assessment, as their daily activity patterns could vary from day to day. They described how it would be useful to have the opportunity to be screened on several occasions to see if the risk had changed, thereby enabling fall risk reduction over the long term. The informants also described that there was a need for more interventions from healthcare professionals to reduce falls by allowing patients to exercise (especially with balance and strength training) at rehabilitation clinics for longer periods of time, rather than attending public gyms. Some informants also thought that the healthcare professional should be more generous with helping aids.

“No, but that you have to do it several times. Several different tests. I think that would also be good. Now you test twice, and then it’s over. Maybe you weren’t really in good shape then.” (Interview 5).

The informants perceived that the screening instrument itself could be more individually adapted to make it possible to increase the degree of difficulty. This could be done, for example, by closing their eyes during the balance test or standing on uneven ground if the initial test was deemed too easy for the individual in question. In terms of development, the informants described that the screening instrument could be used at annual health check-ups, and could prompt the patient’s referral to an appropriate care provider if high fall risk was uncovered. The informants also felt that the screening instrument could help the physiotherapist in assessing the informant’s function and needs.

“Yes, at a doctor’s appointment, for example. When you go to the doctor for an annual check-up, then they do that, a quick little test to start prevention.” (Interview 1)

## Discussion

This focus group interview study gave us a better understanding of how older adults perceive fall risk in general and fall risk screening. The informants shared similar experiences of having been tested with the FIF tool, but based on different experiences and backgrounds, they had both similar and diverging perspectives on falls and screening. Their experiences revealed both the benefits of fall risk screening, but also some concerns. As far as we know, there are no previous studies examining patients’ experiences of being screened for fall risk.

Based on the category *Screening may motivate older adults to act but can also create a false sense of security*, the informants expressed that despite injurious falls being difficult to avoid, aids and various strategies could assuage their concerns about falling and reduce the risk of falls. In another study that involved interviewing older women with osteoporosis, the informants described how they had created various strategies from balance training to managing their daily activities and worries to reduce fall risk [26]. This is also consistent with another study indicating that perceived control is important for people with concerns about falling [27].

Based on the interviews, the informants explained that *Self-sufficiency is affected by the screening result and level of control over the environment* and expressed the importance of being involved in decisions about suggested interventions if the screening test showed an increased risk of falls. Consistent with this, another previous study of participants with Parkinson’s disease observed that individual influence is related to one’s own self-efficacy and self-competence [28]. Another study found that people with a low physiological fall risk but who were still concerned about falling had an increased risk of injurious falls [29]. It is, however, likely that the benefits of screening still outweigh the risks that any false sense of security might involve. There is likely some disagreement about various concepts within our participants since some of the informants described how screening with the FIF tool could create a false sense of security while some describe a feeling of control trough preventive strategies.

The informants described how they perceived falls as highly complex. One of the reasons for this complexity, according to the informants, was the surrounding indoor and outdoor environment, which lies outside of their control. A similar insight and agreement with the participants thoughts about the environmental hazards/barriers was reported in another qualitative study on balance training, in which women with osteoporosis described how environmental factors affected their fall-related concerns [30]. A meta-synthesis of older adult’s experiences with falls highlighted that some people tend to attribute the cause of falls to external factors beyond their control [31]. Previous studies also reinforce the informants’ perception that falls are complex and fall prevention measures need to be individually tailored according to the patient’s physical status and the surrounding environment [32].

Based on the category *Easy to perform and helps to facilitate a discussion with the healthcare professional*, the informants perceived the questions in the FIF tool as being easy to answer. In an earlier study, we investigated the possibility of replacing the balance test in the FIF-tool with a question about the perception of balance ability [33]. The results from this study indicate that physical tests are easier to remember than such questions. In another study, participants with Parkinson’s disease described how balance affected many aspects of their lives—emotionally, physically, and socially. This may also explain why the balance test was perceived as being the most important and remembered part of the FIF tool [34]. The informants explained that it was meaningful to receive feedback on the test results but that it was important to get support from their healthcare provider if they had a high risk of falls. In the study of people with Parkinson’s disease, the participants expressed the importance of their caregivers tailoring the support to their symptoms, expectations, and past experiences in order to be motivated [34].

In the final category *“Ideas for how the FIF tool could be used in healthcare”*, the informants described a desire for the screening to be recurring and more individualized. The current guidelines for fall-risk prevention in community-living older adults recommend a primary screening to identify people at increased risk of falls; and if there is an increased risk, the care provider should continue with an in-depth examination and an individual assessment of the patient’s needs [35]. The focus of screening is, therefore, on quick-to-perform and easy-to-use tools. Screening can help older adults and healthcare staff recognize the risk of falls and take precautionary measures [31]. One idea, described by the informants, was how the FIF tool could facilitate assessment for physiotherapy referral if it were implemented at annual health check-ups. This raises the possibility of testing the screening instrument in new healthcare contexts, with a focus on the healthcare providers’ perspectives on the conditions for implementing the FIF tool in primary healthcare. Patients’ influence has been used to assess the quality of care [11, 12], and our study indicates that patients’ input at an early stage in implementation is just as important. However, it must be taken into consideration the point of view that the participants may have versus what is shown in research. We know that the participants describe the lack of control over their environment as a reason as to why they fall. There is a disagreement from research since we know that a high risk of falling is multidimensional and is caused primarily by imbalance and other risk factors [14].

A recent systematic review suggests that older adults frequently feel unable to take preventive actions against falls. Their beliefs and knowledge become barriers due to social stigma, denial of falls risk, the misconception that falls prevention is for older, more frail people and negative attitudes towards the intervention and its effect [36].

### Strengths and limitations

We assessed the trustworthiness of our study in terms of credibility, dependability, confirmability and transferability [18]. One strength in terms of credibility was the detailed description of the analytical process and that, in addition to the first author, two experienced qualitative researchers were also involved in the data analysis process.

Regarding dependability, interviews were conducted until both the first and the second author experienced saturation [37]. Saturation was reached when the aim of identifying differences and similarities based on the informants’ experiences of having tested the FIF tool were not seen to change the codes, the categories, or the final theme analyzed from the data material [37]. In accordance with confirmability, almost all authors are physiotherapists and were aware that their pre-understanding of both the clinical environment and the topic affected their analysis of the data material.

Another strength of the study is the focus on achieving a sense of security for the informants. This was done by conducting the interviews on a familiar location that was well-known to the informants and would allow the discussions to have an open atmosphere, further facilitating the discussions during the interviews. The choice of primary care clinics at two different locations in Sweden with different socioeconomic conditions increases the study’s transferability, and gave a variety of participants from different backgrounds, which in turn may have provided a broader picture with varying opinions.

One arguable weakness could be the handling of the authors’ subjectivity and pre-understanding, as they were all familiar with the topic discussed during the interviews. Regardless of whether the authors who carried out the data analysis attempted to be objective and compared their interpretations with one another, the degree of subjectivity will affect their interpretation of the analysis process. In this study, where the material was primarily manifest, this should, however, not affect the results to any significant degree. Another arguable weakness is that the informants needed to be able to speak Swedish and would have sought care during the inclusion period, leading to some participants and their perspectives being excluded. A further weakness of the study is that two interviews included only a small number of participants, and that there was a difference in the time it took between screening and interviews among the participants. However, we judged that at least two informants in an interview would be sufficient to contribute valuable information about the patient experience of being screened using the FIF tool. The difference in time may have influenced their recall of the screening process, however, it made it possible for us to observe any sustained behavioral changes or impacts on the informants’ lives that occurred over time as a result of the screening. Another limitation is that the sample included participants coming to a rehab center, of which three participants participated in an exercise class. This may have affected the results of the present study such a way that participants may have had a higher awareness of falls risk and less concerns about falling than the general older population. The FIF tool is not widely used due to its recent development, which can be considered a limitation. However, earlier research demonstrates that the FIF tool has high predictive values for predicting falls, indicating its potential usefulness as screening tool [4, 8].

## Conclusions

Overall, our findings suggest that older adults consider screening for falls to be meaningful and an important fall prevention measure. The results emphasize the need for continued support and assessment to older adults at high risk of injurious falls, and to actively involve patients in the planning of their care. Furthermore, our findings highlight that a low fall risk result may create a false sense of security, which should be kept in mind when communicating the results of the FIF tool assessment. The informants perceived that it was unproblematic to be screened with the FIF tool and proposed that the tool could be used at the annual health check at the health center to screen for fall risk.

## Figures and Tables

**Table 3:**
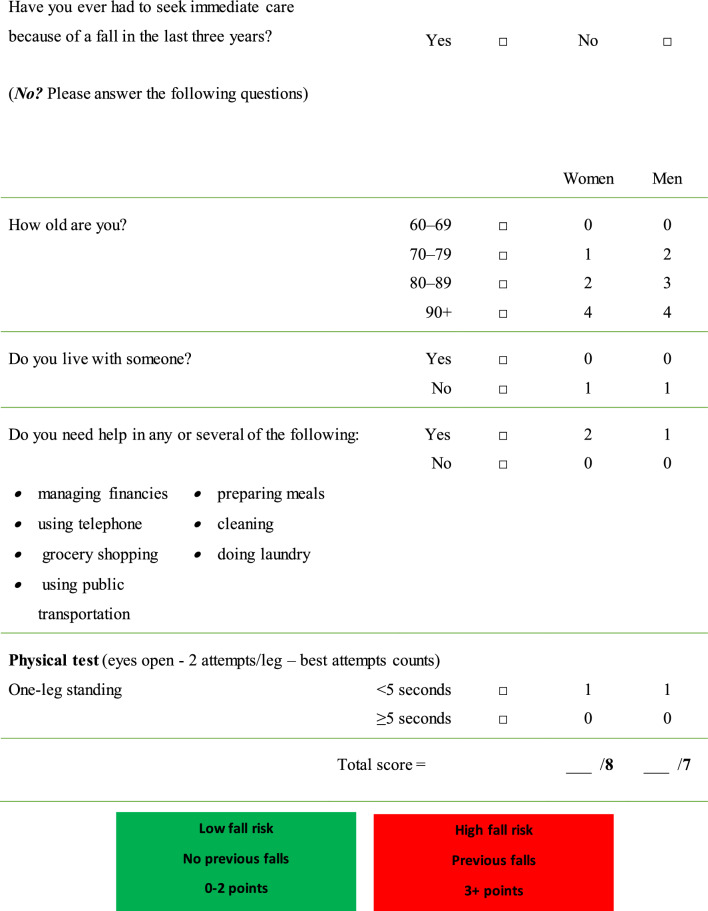
First-time Injurious Falls–FIF screening tool

## Data Availability

The data that support the findings from this study are not publicly available due to the permission given from the Swedish Ethical Review Authority.

## Appendix

See Tables 3, 4.

**Table 4:** Semi-structured interview guide

Description of the background to the study	
1. What do you associate with screening?	
2. What do you think about when I say injurious falls?	
3. How did you find testing the FIF tool with your healthcare provider?	
4. Were there any questions that were hard to answer in the FIF tool?	
5. Did you feel comfortable answering the questions included in the FIF tool? Why/why not?	
6. Were there any questions that were unclear in the FIF tool?	
7. How did you find performing the balance test in the FIF tool?	
8. Did you feel confident when testing the FIF tool? Why/why not?	
9. What do you associate with fall risk?	
10. What were your perceptions of finding out the results from the FIF tool? Regardless of whether the result was a low or high fall risk	
11. How do you feel about getting an idea of your potential risk of an injurious fall in the future?	
12. To what extent do you feel that the test results from the FIF tool are consistent with your own perceptions? If you do not feel that the test results from the FIF tool are consistent with your own perceptions—how do you feel about it?	
13. Do you have expectations about actions or follow-up after having received your results?	
14. Have you changed anything in your everyday life or in your home environment after receiving your FIF tool results?	
15. Do you have any suggestions for improving the FIF tool?	
Do you have any other thoughts or comments that you would like to mention?	
